# Relatively low plasma cortisol levels in parturients are associated with epidural-related maternal fever

**DOI:** 10.1080/07853890.2025.2534086

**Published:** 2025-07-20

**Authors:** Chenxi Zhang, Qinjun Chu, Tao Wang, Hailong Bing, Lihui Bai, Liwei Sun, Dongqing Zhang, Jie Wang, Li Li, Qin Zhou, Zhengyuan Xia, Xiaogao Jin

**Affiliations:** ^a^ Department of Anesthesiology and Perioperative Medicine, Zhengzhou Central Hospital Affiliated to Zhengzhou University, Zhengzhou, P.R. China; ^b^ Department of Anesthesiology, The Third Affiliated Hospital of Zhengzhou University, Zhengzhou, China; ^c^ Department of Obstetrics and Gynecology, The Maternal and Children Hospital of Zhengzhou, Zhengzhou, China; ^d^ Department of Obstetrics and Gynecology, Zhengzhou Center Hospital Affiliated to Zhengzhou University, Zhengzhou, China; ^e^ Department of Anesthesiology, Affiliated Hospital of Guangdong Medical University, Zhanjiang, China; ^f^ Department of Anesthesiology, The Second Affiliated Hospital of Guangdong Medical University, Zhanjiang, China

**Keywords:** Adrenal gland, cortisol, epidural analgesia, inflammation, maternal fever

## Abstract

**Background:**

Epidural analgesia (EA) may suppress the stress response or cortisol production during vaginal delivery by blocking pain transmission. However, there is no evidence that epidural analgesia contributes to epidural-related maternal fever (ERMF) by inhibiting the hypothalamic pituitary adrenal (HPA) axis.

**Objective:**

This study aimed to evaluate the relationship between maternal cortisol levels and ERMF during vaginal delivery.

**Study design:**

Prospective cohort study.

**Methods:**

The venous blood samples were collected from full-term pregnant women undergoing vaginal delivery and categorized into control group (<37.5 °C without EA), normal group (<37.5 °C with EA), mild group (37.5–37.9 °C with EA) or fever group (≥38 °C with Epidural analgesia (EA)). Then, plasma cortisol and pro-inflammatory cytokine levels (IL-6, TNF-α, IL-2 and IFN-γ) were assessed to determine the impact of EA on the stress response. We also detected hormones related to the HPA axis, including CRH and ACTH plasma concentration. Additionally, general adrenal function was evaluated by measuring renin, aldosterone, testosterone, and DHEA-s concentrations in the maternal venous blood. Clinical evidence of adrenal dysfunction was also investigated using medical records.

**Results:**

Epidural analgesia induced high levels of cytokines IL-6 and TNF-α in all parturients, but ERMF was observed only in those with lower cortisol levels. Correlation analysis revealed a positive relationship between IL-6 (or TNF-α) and cortisol concentration ratio and maximum maternal temperature. Elevated ACTH, β-endorphin and αMSH levels were observed in parturients with ERMF, indicating primary adrenal dysfunction. Medical records showed no significant differences in symptoms, signs, or laboratory results related to adrenal function among the four groups.

**Conclusion:**

There was a relationship between relatively low maternal cortisol levels and ERMF.

**Trial registration number:**

ChiCTR2200058359 obtained on April 7, 2022.

## Introduction

Epidural analgesia administered during vaginal delivery is known to cause fever (temperature ≥38 °C) in approximately 15–25% of parturients [1,2], which is also referred to as epidural-related maternal fever (ERMF), and has also been observed following the use of a combination of epidural and spinal analgesia [3,4]. Recent studies have raised concerns about the safety of epidural analgesia due to its potential association with autism spectrum disorder in offspring [5–7]. These findings highlight the importance of understanding the mechanisms underlying ERMF and validating the safety of epidural analgesia. In addition, maternal immune activation resulting from ERMF may lead to fetal brain injuries during epidural analgesia [8,9]. Therefore, further research on the mechanism of ERMF is important for developing more efficient strategies to ensure patient safety.

Current literature has demonstrated that ERMF can be triggered by systemic inflammation without infection in parturients, characterized by elevated levels of pro-inflammatory cytokines, such as IL-6, TNF-α, and IL-1β [10,11]. Despite previous suggestions that the induction of local anesthesia may damage the placenta and promote the production of pro-inflammatory cytokines during ERMF [12,13], the exact mechanism of underlying ERMF remains elusive. In clinical settings, various approaches have been employed to reduce the incidence of ERMF, including intermittent administration of epidural local anesthetics, reducing local anesthetic concentration, decreasing epidural opioid dosage and additional steroid administration [6,14,15]. Among these, administration of high-dose steroids has been found to be effective in lowering the incidence of ERMF[16,17], indicating a possible link between ERMF and changes in adrenal function.

Although evidence suggests that additional steroid administration can reduce the incidence of ERMF, specific changes in the hypothalamic-pituitary-adrenal (HPA) axis during epidural analgesia are not well understood. Based on the mechanism of epidural analgesia, it is believed that the HPA axis is suppressed following neuraxial blockade by local anesthetics; however, surprisingly, there have been no investigations on the functional changes in the HPA axis during epidural analgesia. Additionally, it is known that the placenta plays a vital role in regulating maternal HPA axis activity by producing significant amounts of CRH and ACTH during pregnancy, and the maternal adrenal gland produces high-levels of cortisol in response to elevated ACTH levels [18], resulting in two to three-fold increases in total maternal plasma cortisol during pregnancy. Thus, to protect the fetus, the placenta produces 11 beta-hydroxysteroid dehydrogenase type 2 (11βHSD2) to inactivate such high level cortisol [18]. The intricate nature of the maternal stress response throughout pregnancy leads us to hypothesize that alterations in the maternal stress response during epidural analgesia might contribute to the occurrence of ERMF. Hence, this study was designed to investigate the relationship between labor epidural analgesia and the cortisol response, as well as the inflammatory response, and to further explore the mechanism underlying ERMF.

## Methods

### Parturitions included in this study

This prospective cohort study was carried out at Zhengzhou Central Hospital Affiliated to Zhengzhou University from April 10, 2022, to July 20, 2023. The Medical Ethics Committee of Zhengzhou Central Hospital Affiliated to Zhengzhou University approved this study (202238). In the Chinese Clinical Trial Registry, registration number ChiCTR2200058359 was obtained on April 7, 2022. This study was also conducted in accordance with the Declaration of Helsinki. The Maternal and Children’s Hospital of Zhengzhou provided guidance in epidural analgesia procedure and temperature measure.

The study participants were singleton pregnancies, aged 18–45 years, regular contractions, cervical dilation of 3 cm or more, ASA physical status I or II, and were at full term. Patients with spinal diseases, underlying conditions such as hyperthyroidism or hypothyroidism, gestational diabetes mellitus, HIV infection, high-risk pregnancies (i.e. severe preeclampsia/eclampsia, placental abruption, placenta previa), and infected with Covid-19 were excluded. An attending obstetrician would determine those who could deliver vaginally and enter the labor room by evaluating regular uterine contractions and obstetric indications. Participants were also excluded when Cesarean section was performed during the investigation. Written informed consents were obtained for all eligible participants before blood samples and placenta were collected.

Four groups were established from the included patients including the control, normal, mild, and fever groups, based on two criteria: whether they received epidural analgesia and their maximum temperature after EA. Patients who did not have fever (*T* < 37.5 °C) and received no epidural analgesia were included in the control group. However, none of the patients exhibited a fever (*T* > 37.5 °C) without receiving epidural analgesia. Those who received epidural analgesia were divided into three groups according to their recorded maximum temperature during labor: normal group (*T* < 37.5 °C), mild group (37.5–37.9 °C), and fever group (*T ≥* 38 °C).

### Study design and sample size calculation

This study was a prospective cohort study. The primary endpoint was the maternal plasma cortisol level immediately after delivery. The sample size was determined based on our preliminary results on maternal plasma levels during vaginal delivery. The sample size calculation suggested the need for at least 10 patients in each group based on the following parameters [19]: α = 0.05, *Z*_α_ = 1.96, power = 80%, *Z*_β_ = 0.84, expected standard deviation = 25 µg/dl, and accepted effect size = 30 µg/dl [20].

### Epidural analgesia protocol for vaginal delivery

Epidural analgesia for vaginal deliveries was performed as previously described [3]. Briefly, the epidural procedure was performed as follows: With the parturient in lateral decubitus position, an 18-gauge Tuohy needle was inserted at the L3–L4 interspace. Following successful puncture, a catheter was threaded 4–5 cm into the epidural space. A test dose of 3 mL 1.5% lidocaine (10 mg/mL) containing epinephrine 0.5 μg/mL (1:200,000) was administered through the catheter to exclude intrathecal or intravascular placement. After confirming negative aspiration and absence of complaint during a 5-minute observation period, an initial bolus of 10 mL solution containing 10 mg ropivacaine (0.1%) and 5 μg sufentanil was delivered. Continuous analgesia was maintained *via* an electronic PCA pump programmed with: 1) background infusion at 8 mL/h (ropivacaine 0.08% + sufentanil 0.4 μg/mL); 2) demand bolus of 8 mL; 3) 15-minute lockout interval; 4) maximum hourly dose of 32 mL. The maternal temperature was measured every 1/2 h throughout labor using a tympanic probe (ThermoScan3, Infrared ear thermometer, Braun, Germany).

### Blood sample and placenta retrieval

Peripheral venous blood samples (approximately 5 ml each) were collected from the antecubital fossa vein of parturients approximately immediately after delivery in a purple vacuum tube (Sanli, Hunan, China) containing ethylenediaminetetraacetic acid (EDTA) to prevent anticoagulation. Then, the tube was gently inverted 6–8 times to ensure thorough mixing of the contents, centrifuged at 3,000 rpm for 5 min, and the resulting supernatant or plasma was aspirated into several 1.5 ml Ep tubes and stored in a refrigerator at −80 °C until subsequent use.

### Determination of the plasma concentration cortisol and pro-inflammatory cytokines

Cortisol concentrations were determined by ELISA(Cloud-Clone, Wuhan, China). The plasma levels of pro-inflammatory cytokines, including IL-6, TNF-α, IL-2 and IFN-γ, were measured using the flow cytometry fluorescence method. The human Th1/Th2 subgroup detection kit was purchased from Jiangxi Saiji Biotechnology Company Limited (Nanchang, China). Flow cytometry was performed using BriCyte E6 apparatus (Mindray, Shenzhen, China).

### Determination of the maternal plasma concentration of CRH, ACTH, β-endorphin, and αMSH

The levels of CRH (Human CRH Corticotropin Releasing Hormone) were determined using an ELISA Kit (Elabscience, E-EL-H1144c, Wuhan, China), along with ACTH (Adrenocorticotropic Hormone) measured by the ELISA Kit (Cloud-Clone, CEA836Hu, Wuhan, China), αMSH (Alpha-Melanocyte Stimulating Hormone) was analyzed using an ELISA Kit (Cloud-Clone, CEA239Hu, Wuhan, China), and β-endorphin was assessed by the ELISA Kit (Cloud-Clone, CEA806Hu, Wuhan, China). For each plasma sample, appropriate dilutions were prepared and incubated in the respective wells at 37 °C for 1 h. Subsequently, an enzyme-labeled secondary antibody was added, and the wells were washed five times. The substrate solution was then added in each well to induce color development. Upon reaching the desired color intensity, a stop solution was added and the absorption at 450 nm was measured using a plate reader (SpectraMax i3x, Molecular Devices, Silicon Valley, USA). Finally, hormone concentrations were calculated based on the standard curve.

### Full evaluation of maternal adrenal function (renin, aldosterone, DHEA-S, testosterone)

The aldosterone and renin levels in maternal plasma were measured using a chemiluminescent immunoassay (CLIA) with the LIAISON Aldosterone kit and LIAISON Direct Renin kit (DiaSorin Inc., Stillwater, Minnesota, USA), respectively, using the LIAISON XL fully automated immunoassay analyzer (DiaSorin Inc., Stillwater, Minnesota, USA). In addition, testosterone and DHEA-S levels were measured using a paramagnetic particle, chemiluminescent immunoassay. For this purpose, the Access Testosterone kit and Access DHEA-S kit (Beckman Coulter, USA) were used, and immunoassays were conducted using the UniCel DXI 800 Access Immunoassay System (Beckman Coulter Inc., USA).

### Investigation of adrenal-insufficiency-related evidences

We collected data on symptoms and signs associated with adrenal insufficiency from medical records. If the patients reported no experience of these symptoms during pregnancy, they were regarded as absent. The symptoms of adrenal insufficiency encompassed fatigue, salt craving, anorexia, postural dizziness, and joint and muscle aches [18]. The signs of adrenal insufficiency include weight loss, abdominal ­discomfort, loss of axillary and pubic hair, and increased pigmentation in the skin and mucous ­membranes [18].

Laboratory results related to adrenal insufficiency were analyzed in the four groups. These results were obtained on the first day after the patients were admitted and planned to undergo natural labor. Laboratory tests included measurements of plasma electrolyte concentrations (Na^+^, K^+^, Ca^2+^, Cl^–^), fasting plasma glucose levels, urine pH value, urine specific gravity, hemoglobin (Hb) concentration, and percentages and counts of blood cells (lymphocytes and eosinophils) [18].

### Statistical analysis

Data are presented as the mean ± standard deviation (SD). The Kolmogorov–Smirnov test was used to assess the normality of the distribution of continuous data. One-way analysis of variance (ANOVA) was used to compare the four groups followed by Tukey–Kramer post hoc testing. A correlation analysis was performed between the maximum maternal temperature and the ratio of IL-6 or TNF-α to cortisol. The chi-square test was used to compare categorical variables, and Fisher’s exact test was used as deemed appropriate. In this study, statistical significance was set at a two-sided *P* value of ≤0.05.

## Results

### Characteristics of the parturients from the four groups

**Figure 1.**
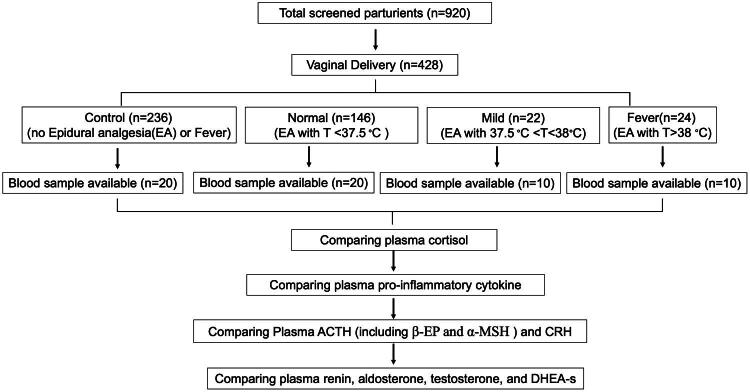
Study flow chart. This study has screened 920 parturients and 428 parturients of them delivered through vagina. The parturients with vaginal delivery were divided into four groups: control (*n* = 236), normal (*n* = 146), mild (*n* = 22), fever (*n* = 24). After selection by inclusion criteria, exclusion criteria, and blood sample available, a total of 60 parturients were finally included in this study to detect plasma cortisol, cytokine, and related hormones (control (*n* = 20), normal (*n* = 20), mild (*n* = 10), fever (*n* = 10)).

In this study, 60 parturient patients were included in the analysis, and their blood samples were successfully collected through their approval. The number of parturients in each group is shown in the study flow-chart (Figure 1). Patients in the Fever group had a longer hospital stay than those in the normal group. These results suggest that ERMF might lead to longer hospital stays (Table 1).

**Table 1.** Characteristics of the parturient patients.

Characteristics	Control (*n* = 20)	Normal (*n* = 20)	Mild (*n* = 10)	Fever (*n* = 10)	*F*/χ^2^	*P*
Age (years)	29.85 ± 3.731	28.15 ± 3.468	27.30 ± 3.831	27.40 ± 2.836	1.746	0.1681
BMI (kg/m^2^)	26.62 ± 3.208	26.94 ± 2.699	25.09 ± 3.045	26.62 ± 1.212	1.049	0.3783
MAP (mmHg)	89.90 ± 8.317	92.62 ± 6.440	85.50 ± 7.106	87.73 ± 5.152	2.577	0.0627
HR (bpm)	83.30 ± 6.300	81.75 ± 5.087	84.10 ± 4.095	83.30 ± 3.368	0.5683	0.6382
Gestational age (weeks)	39.39 ± 1.346	39.32 ± 0.7932	38.64 ± 1.467	39.20 ± 0.8417	1.057	0.3746
Parity history					2.927	0.4030
0	11	14	8	8		
1	9	6	2	2		
FHR before analgesia (bpm)	141.5 ± 9.730	142.4 ± 11.43	138.8 ± 7.857	146.8 ± 6.579	1.212	0.3139
Lateral episiotomy (*n*%)	7(35%)	6(30%)	4(40%)	3(30%)	0.3750	0.9454
Apgar score (5 min)	10.00 ± 0.000	10.00 ± 0.000	10.00 ± 0.000	10.00 ± 0.000		1
Apgar score (10 min)	10.00 ± 0.000	10.00 ± 0.000	10.00 ± 0.000	10.00 ± 0.000		1
Fetal sex					1.989	0.5748
Male	10	6	5	4		
Female	10	14	5	6		
Fetal weight (g)	3265 ± 501.5	3287 ± 397.9	3152 ± 382.9	3290 ± 337.0	0.2575	0.8557
Length of hospital stay (days)	4.300 ± 1.559	4.200 ± 1.281	5.300 ± 1.059	5.600 ± 1.075#	3.766	0.0156^#^

# *p* < 0.05 when compared to the normal group.

### Plasma cortisol concentration increased in non-febrile pregnant women following epidural analgesia but decreased in febrile women with epidural analgesia

**Figure 2.**
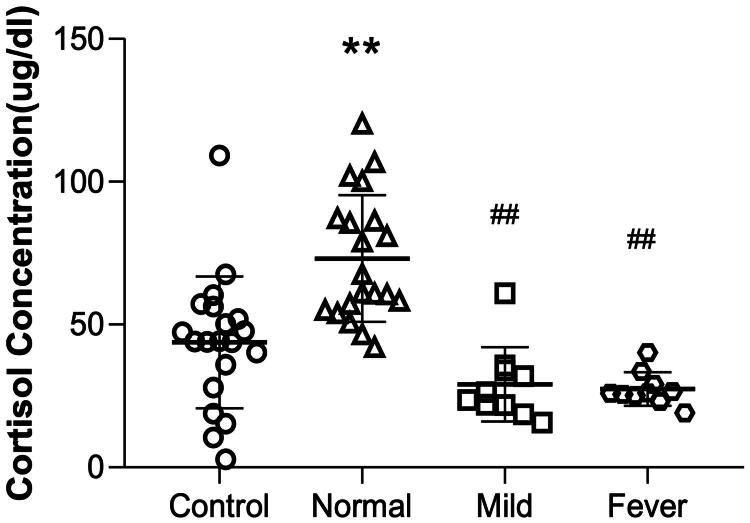
Plasma cortisol concentration of the four groups. The plasma cortisol concentrations were 43.76 ± 23.10, 73.12 ± 22.20, 29.04 ± 13.00, 27.43 ± 5.83 for control, normal, mild, and fever groups, respectively. **p < 0.01 compared to the control group, ^##^*p* < 0.01(^#^*p* < 0.05) compared to the normal group, *N* = 20 in control and normal groups, *N* = 10 in mild and fever groups. Data shown are the mean ± SD.

We observed an increase in maternal plasma cortisol concentration in the normal group, while cortisol concentration decreased in the mild and fever groups when compared to the control group (Figure 2). Moreover, the mild and fever groups had a lower cortisol concentration than the normal group. Taken together, these results suggest a potential association between maternal cortisol concentration and ERMF.

### Epidural analgesia-induced IL-6 and TNF-α production in parturitions

**Figure 3.**
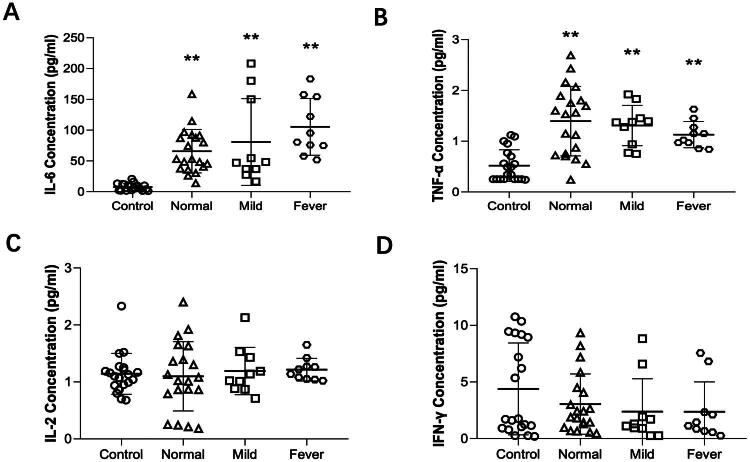
The changes of IL-6, TNF-α, IL-2 and IFN-γ concentrations in the parturients. **p < 0.01 compared to the control group, *N* = 20 in control and normal groups, *N* = 10 in mild and fever groups. Data shown are the mean ± SD.

We observed an increase in the concentration of maternal plasma IL-6 and TNF-α in the normal, mild and fever groups when compared to the control group, while no significant differences were observed among the normal, mild and fever groups (Figure 3A,B). No significant differences were observed in IFN-γ and IL-2 concentrations among the four groups (Figure 3C,D). These findings suggest that epidural analgesia induces systemic inflammation in parturient patients during vaginal labor.

### Positive correlations between the maximal maternal temperatures and IL-6/cortisol or TNF-α/cortisol

**Figure 4.**
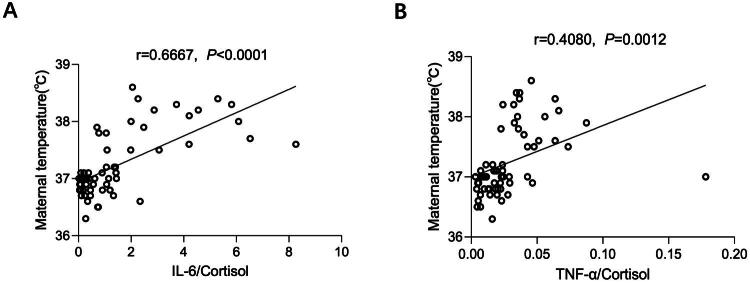
The correlations between the maximal maternal temperatures and IL-6 (TNF-α)/cortisol. A: the correlation analysis of maximal maternal temperature and the ratio of IL-6 over cortisol. *r* = 0.6667 (*p* < 0.0001). Total *N* = 60. B: the correlations between the maximal maternal temperatures and TNF-α/cortisol. *r* = 0.4080 (*p =* 0.0001). Total *N* = 60.

To evaluate the role of cortisol in epidural-related maternal fever (ERMF), we used the highest maternal temperature recorded during labor as a measure of systemic inflammation. Furthermore, we analyzed the correlation between peak temperature and the ratios of interleukin-6 to cortisol (IL-6/cortisol) or tumor necrosis factor-alpha to cortisol (TNF-α/cortisol). The findings revealed a direct correlation between the highest maternal temperature and the ratio of IL-6 or TNF-α to cortisol (depicted in Figure 4A,B), implying that cortisol might counteract the effects of IL-6 or TNF-α, thereby preventing the onset of fever during epidural analgesia. Moreover, reduction in cortisol levels may contribute to the incidence of ERMF.

### Stress hormones from the pituitary gland are elevated in febrile women who underwent epidural analgesia

**Figure 5.**
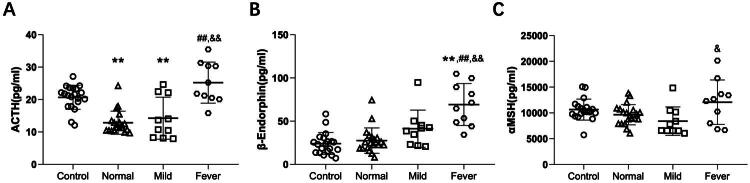
Plasma ACTH, β-EP and α-MSH concentrations in the four groups. Data are shown as mean ± SD. ^##^*p* < 0.01 compared to normal group, *p < 0.05 and **p < 0.01 compared to the control group, ^&^*p* < 0.05 and ^&&^*p* < 0.01 compared to mild group. *N* = 20 in control and normal groups, *N* = 10 in mild and fever groups.

To investigate whether cortisol reduction is a primary or secondary change in the adrenal gland, the maternal plasma concentrations of ACTH, β-EP, and α-MSH were detected during vaginal delivery. The results showed that the Normal and Mild groups had lower ACTH levels than the control group, while the fever group had higher ACTH levels than the normal or mild groups (Figure 5). Similarly, β-EP levels in the fever group were higher than those in the control, normal and mild groups. Additionally, α-MSH concentrations also increased in the fever group compared to those in the mild group. Collectively, these findings suggest that cortisol reduction in ERMF parturients could be one of the primary changes in the adrenal gland.

### No differences in plasma CRH among all the four groups

**Figure 6.**
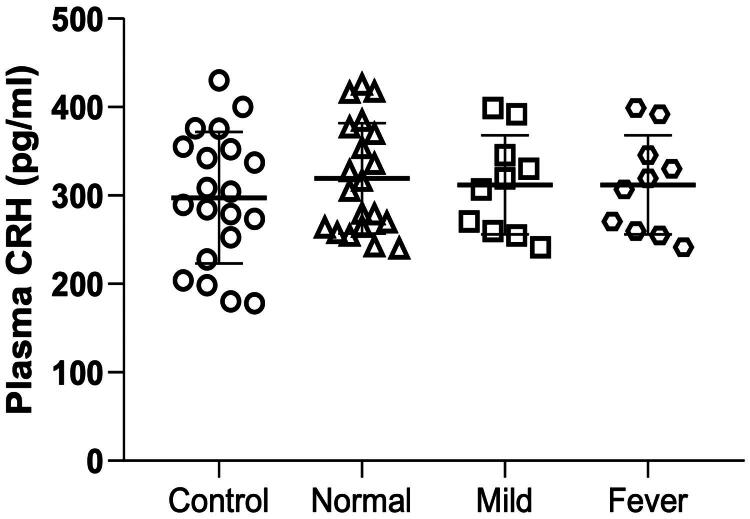
Plasma CRH concentration in the four groups. Data are shown as mean ± SD. No significant differences were found between the four groups. *N* = 20 in control and normal groups, *N* = 10 in mild and fever groups.

The evaluation of maternal plasma CRH concentration revealed that despite the decrease in placental CRH expression in the fever group, there were no significant differences in maternal plasma CRH levels among the four groups (Figure 6). This indicated that parturients with ERMF had a normal concentration of CRH from the maternal hypothalamus.

### Adrenal function assay showed aldosterone (ADL) reduction in the febrile women with epidural analgesia

**Figure 7.**
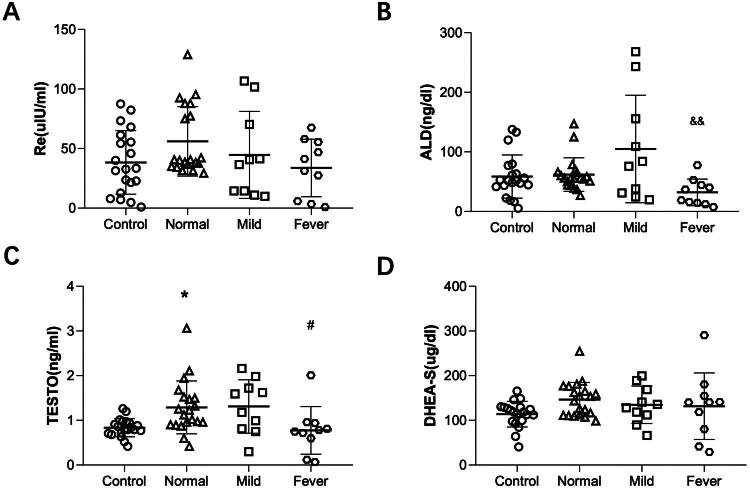
The concentration of plasma renin, ALD, TESTO and DHEA-S in the four groups. *p < 0.05 compared to the control group, ^#^*p* < 0.05 compared to the normal group, ^&&^*p* < 0.01 compared to the mild group. *N* = 20 in control and normal groups, *N* = 10 in mild and fever groups. Data are shown as mean ± SD.

Our findings indicate that pregnant women with ERMF experience a relative primary adrenal insufficiency in cortisol production. The adrenal gland in ERMF patients may also produce aldosterone, dehydroepiandrosterone (DHEA), testosterone, adrenaline, and norepinephrine. However, the present study did not detect these hormones because significant variations in adrenaline and norepinephrine levels based on the environment. Instead, we focused on measuring DHEA-S, the sulfated metabolite of DHEA that is more stable [21]. Renin is produced by juxtaglomerular cells of the kidney and participates in the renin-angiotensin-aldosterone system to regulate blood pressure. Plasma renin level was also measured to evaluate whether the changes in aldosterone were primary or secondary. Our results showed no significant differences in plasma renin, testosterone, and DHEA-S levels among the four groups. However, the fever group exhibited lower aldosterone concentrations than the mild group. These results indicate that the reduction in aldosterone could be primary, as there were no differences in renin levels among the groups (Figure 7). Aldosterone and cortisol shares the same substrate and some synthases [22]. Aldosterone and cortisol synthesis may be inhibited in parturients with ERMF.

### No evidences were found for adrenal-insufficiency-related symptoms, signs, and laboratory results in the four groups

Although maternal plasma cortisol decreased in ERMF parturient women, no significant differences were observed in adrenal insufficiency-related symptoms and signs in the four groups (Supplemental Table 1). Laboratory results related to adrenal insufficiency may be more sensitive than symptoms and signs associated with adrenal insufficiency (Supplemental Table 2). Therefore, we compared the laboratory results of the four groups. Despite the presence of a relatively low cortisol level in the ERMF group, no significant differences were found in the adrenal-insufficiency-related laboratory results among the four groups.

## Discussion

This study found a relationship between ERMF and relatively low maternal plasma cortisol levels. The parturients with relatively low adrenal levels experienced epidural-related maternal fever (ERMF) during labor. And, correlation analysis indicated a positive relationship between maximal temperature and the ratio of IL-6 or TNF-α to cortisol, implying that cortisol play a crucial role in counteracting the febrile effects of pro-inflammatory cytokines during labor under analgesia [23]. Further investigation indicated the decrease of maternal cortisol during ERMF may involve in the primary adrenal change. The medical records revealed that the parturients with comparatively low adrenal levels during ERMF did not manifest any typical clinical symptoms of adrenal insufficiency.

### Relationship between relatively low cortisol level and ERMF

Although our data showed that fever parturients with high ACTH and low cortisol levels may have primary adrenal insufficiency, the incidence of ERMF is up to 10–20% which exceeds the prevalence of adrenal insufficiency in pregnancies (9.6/100,000) [18]. Moreover, the diagnostic criteria for adrenal insufficiency during pregnancy suggest that a morning cortisol level below 83 nmol/L (3 μg/dL) could be assessed as significance [18,24]. However, this cutoff value is too low for pregnant women, and in the present study, all the investigated parturients in the four groups had cortisol levels above 10 μg/dL[6,24]. According to Jung et al. total plasma cortisol levels should be >600 nmol/L (21.7 μg/dL) in the third trimester of pregnancy, compared to approximately 300 nmol/L (10.8 μg/dL) in non-pregnant women not taking oral contraceptive pills [12]. In our study, the mean cortisol level in the fever group was 25.59 μg/dL, indicating that the total plasma cortisol levels in both the mild and fever groups were within the normal range. There was no clinical evidence indicating the presence of symptoms, signs, or laboratory results related to adrenal insufficiency. In this study, adrenal insufficience could not be diagnosed without a stimulation test. Therefore, the relatively low cortisol level in the ERMF parturients may be a temporary phenomenon, or may be caused by the greater degradation of cortisol during inflammation. Further studies are necessary to confirm whether there is a relative adrenal insufficiency in ERMF parturients.

### Epidural analgesia induced systemic inflammation during natural delivery

All women receiving epidural analgesia displayed elevated levels of pro-inflammatory cytokines in their plasma compared to those who did not undergo epidural analgesia. These findings suggest that factors such as epidural puncture, catheterization, and local anesthetics may contribute to the increased production of pro-inflammatory cytokine [2]. Epidural puncture and catheterization may have a weak association with ERMF, as patients undergoing Cesarean section under epidural anesthesia experience fever less frequently than those undergoing labor [25]. Local anesthetics have been shown to induce monocytes to produce pro-inflammatory cytokines [13,26,27], and can also cause placental injury, leading to macrophages producing an inflammasome and triggering further pro-inflammatory cytokine production [13]. However, these findings do not fully explain why only a small proportion of parturient women experience ERMF during epidural analgesia. Although elevated pro-inflammatory cytokines may induce maternal fever during epidural analgesia, inhibitory hormones and cytokines such as cortisol, progesterone, and IL-10 are simultaneously upregulated. Parturients receiving epidural analgesia might avoid febrile responses due to sufficient anti-inflammatory mediators to counteract the pro-inflammatory surge. Thus, alterations in maternal plasma cortisol levels could be involved in ERMF.

### Study limitations

This study has several limitations. First, there were no parturients with fever but without epidural analgesia. Therefore, no comparison was performed between fever parturients with epidural analgesia and fever parturients without epidural analgesia. Second, the symptoms and signs were retrospectively collected after identifying blunted adrenal response. These symptoms and signs are non-specific and may often be overlooked by physicians during medical history collection and physical examinations. Finally, we collected the maternal blood samples once immediately after delivery. Maternal blood samples before epidural analgesia and during delivery are necessary to evaluate the effect of epidural analgesia on cortisol production. We couldn’t confirm whether changes in cortisol levels are the cause or the result of fever.

## Conclusion

In this study, relatively low cortisol levels in parturients were found to be associated with ERMF during labor. Further investigations are necessary to confirm whether decreased adrenal function contributes to ERMF.

## Human ethics and consent to participate declarations

The Medical Ethics Committee of Zhengzhou Central Hospital Affiliated to Zhengzhou University approved this study (202238). Informed consent was obtained by all eligible participants who willingly participated in this study in accordance with the Declaration of Helsinki.

## Consent for publication

Not applicable.

## Supplementary Material

Supplemental Material

Supplemental Material

## Data Availability

Data generated in this study are available from the corresponding author upon request.
