# Supplementary material for: Relatively low plasma cortisol levels in parturients are associated with epidural-related maternal fever
Source: Ann Med. 2025 Jul 20;57(1):2534086. doi: 10.1080/07853890.2025.2534086 (PMC12278448; doi:10.1080/07853890.2025.2534086)
Supplement: Supplemental Material [file IANN_A_2534086_SM3073.docx]

**Supplemental table 2. The laboratory results related to adrenal insufficiency in the patients**

|  | **Control(n=20)** | **Normal(n=20)** | **Mild(n=10)** | **Fever(n=10)** | **F** | ***P*** |
| --- | --- | --- | --- | --- | --- | --- |
| Na (mmol/l) | 137.4±1.653 | 137.3±1.121 | 135.9±2.040 | 136.4±2.686 | 2.015 | 0.1222 |
| K+ (mmol/l) | 3.864±0.1582 | 3.880±0.2929 | 3.781±0.4744 | 3.986±0.3134 | 0.7983 | 0.5000 |
| Ca2+ (mmol/l) | 2.247±0.1257 | 2.241±0.09920 | 2.290±0.1057 | 2.182±0.08587 | 1.698 | 0.1778 |
| Cl- (mmol/l) | 105.0±1.709 | 105.5±2.549 | 103.8±1.696 | 104.7±1.291 | 1.681 | 0.1814 |
| RBC (10^12^/L) | 3.643±0.3466 | 3.775±0.3785 | 3.642±0.3074 | 3.954±0.2510 | 2.234 | 0.0943 |
| Hb (g/L) | 112.8±10.23 | 117.8±10.12 | 112.8±5.750 | 121.7±9.978 | 2.536 | 0.0659 |
| FBG (mmol/l) | 4.761±0.2125 | 4.838±0.3803 | 4.871±0.1332 | 5.011±0.5171 | 1.288 | 0.2874 |
| Urine pH | 6.625±0.2751 | 6.425±0.4667 | 6.400±0.5164 | 6.350±0.5297 | 1.267 | 0.2946 |
| Urine Specific gravity | 1.015±0.004128 | 1.016±0.004861 | 1.017±0.004216 | 1.015±0.005774 | 0.6315 | 0.5978 |
| Lymphocytes(10^9^/L) | 1.490±0.4518 | 1.788±0.6382 | 1.811±0.4862 | 1.573±0.3815 | 1.499 | 0.2249 |
| Percentage of lymphocytes (%) | 18.78±3.661 | 20.28±4.683 | 18.73±6.237 | 17.48±7.215 | 0.7211 | 0.5436 |
| Eosinophils(10^9^/L) | 0.06050±0.03706 | 0.07050±0.05726 | 0.09600±0.08329 | 0.03600±0.03565 | 2.186 | 0.0998 |
| Percentage of eosinophils (%) | 0.7707±0.4515 | 0.8016±0.5748 | 0.9290±0.8700 | 0.4000±0.3528 | 1.658 | 0.1865 |
