# Supplementary material for: Relatively low plasma cortisol levels in parturients are associated with epidural-related maternal fever
Source: Ann Med. 2025 Jul 20;57(1):2534086. doi: 10.1080/07853890.2025.2534086 (PMC12278448; doi:10.1080/07853890.2025.2534086)
Supplement: Supplemental Material [file IANN_A_2534086_SM3072.docx]

**Supplement Table 1. The adrenal-insufficiency-related symptoms and signs of the patients**

| Symptoms | Control(n=20) | Normal(n=20) | Mild(n=10) | Fever(n=10) | Fisher's | P |
| --- | --- | --- | --- | --- | --- | --- |
| Fatigue(1/2/3/4) | 18/2/0/0 | 16/4/0/0 | 8/2/0/0 | 9/1/0/0 | 1.196 | 0.754 |
| Weight loss(1/2/3/4) | 20/0/0/0 | 20/0/0/0 | 10/0/0/0 | 10/0/0/0 |  | 1 |
| Salt craving(1/2/3/4) | 20/0/0/0 | 20/0/0/0 | 10/0/0/0 | 10/0/0/0 |  | 1 |
| Postural dizziness(1/2/3/4) | 20/0/0/0 | 18/2/0/0 | 9/1/0/0 | 9/1/0/0 | 3.385 | 0.336 |
| Anorexia(1/2/3/4) | 19/1/0/0 | 18/2/0/0 | 8/2/0/0 | 10/0/0/0 | 3.468 | 0.325 |
| Abdominal discomfort(1/2/3/4) | 19/1/0/0 | 20/0/0/0 | 10/0/0/0 | 10/0/0/0 | 2.231 | 0.526 |
| Joint and muscle aches(1/2/3/4) | 19/1/0/0 | 18/2/0/0 | 8/2/0/0 | 9/1/0/0 | 1.556 | 0.669 |
| Hair loss(1/2/3/4) | 20/0/0/0 | 20/0/0/0 | 10/0/0/0 | 10/0/0/0 |  | 1 |
| Skin Pigmentation(1/2/3/4) | 16/4/0/0 | 15/5/0/0 | 6/4/0/0 | 7/3/0/0 | 1.403 | 0.705 |

Note: These symptoms and signs were categorized into four different grades, with Grade 1 indicating no manifestations and no impact on daily activities, Grade 2 representing mild manifestations with slight interference in daily activities that didn't require treatment, Grade 3 denoting noticeable manifestations that required treatment, and Grade 4 indicating severe manifestations that required hospitalization.

Skin pigmentation was also classified into four grades, in which Grade 1 indicated no change in skin pigmentation, Grade 2 indicated increased pigmentation only in exposed skin, Grade 3 indicated increased pigmentation in both the exposed and unexposed areas (i.e., the axilla and inguinal region), and Grade 4 indicated increased pigmentation all over the skin, including oral mucosa.
